# MAIT cells: the end of the beginning?

**DOI:** 10.1111/imcb.12056

**Published:** 2018-06-02

**Authors:** Daniel G Pellicci, Paul Klenerman

**Affiliations:** ^1^ Department of Microbiology and Immunology Peter Doherty Institute for Infection and Immunity University of Melbourne Melbourne VIC 3000 Australia; ^2^ Peter Medawar Building for Pathogen Research Nuffield Department of Medicine University of Oxford South Parks Road Oxford OX1 3SY UK

Mucosal associated invariant T cells (MAIT) are evolutionarily old, but scientifically still quite young. The first descriptions of a human T‐cell population with the canonical T‐cell receptors were 25 years ago (reviewed by Lantz and Legoux^1^). However, it is in the last few years, since the discovery of their specificity, functionality and sheer abundance that interest has really begun to develop and spread into different fields of biology and medicine. This is part of a growing interest in innate‐like or unconventional T cells which includes iNKT cells and other CD1‐restricted populations, as well as subsets of gamma‐delta T cells. The question of how such cells function in health and disease has developed into one of the most exciting areas in immunology, with implications from infections to cancer. This collection of seven Special Feature reviews focused on MAIT cells will help set the scene and connect some of the dots between different parts of this rapidly emerging field.

The Special Feature reviews in this issue start with the historical perspective on MAIT cells – in terms of both their discovery and their evolution – which may give some clue as to their critical and overlapping functions.^1^ This leads naturally on from the cells themselves to what they recognize – a major set of discoveries which has led to an explosion of interest in the field.^2^, ^3^ The development of this cell type and its selection in the thymus is next explored, an area that can give insight into not only MAIT cell biology but that of other related cell types.^4^ The last three reviews look further into the biological roles of these cells. This includes a perspective on the best‐described areas of MAIT cell research – namely antimicrobial defense, based on the specificity of the TCR for the microbially‐derived ligand.^5^ Given their potential for stimulation via “danger” signals, MAIT cells may also be involved more broadly in host defense and inflammation. This aspect is explored in reviews on auto‐immune disease^6^ and antiviral responses.^7^

The emerging picture from these reviews and the original data they describe is of a fascinating cell type, with important protective and also pathological potential. So, the cells are interesting – and in humans highly abundant– but are they important? This perhaps is one of the major next questions for the field. MAIT cells form part of a team with other unconventional T cells, with overlapping targets and effector functions. Which parts of their biology are essential or non‐redundant needs to be better defined. And designing experiments in both animal models and in humans to test these ideas is an important challenge, as these cells appear to be functionally distinct between some species. Another interesting question emerging is whether their role extends beyond that of “emergency” responses, to include more homeostatic functions linked to interactions with commensal bacteria at barrier sites.^8^ Addressing this alternative lifestyle for MAIT cells and related T‐cell subsets will need a different type of experimental approach and clinical study but could be equally important.

At this point, there is now a wealth of data on MAIT cells but many as‐yet‐unanswered questions are posed in each review. We now have many important tools at our disposal to address these questions *in vitro* and *in vivo* and the field is moving into perhaps the next phase of discovery. Certainly, it is not the beginning of the end for defining their real significance – and also for harnessing their functional potential – but perhaps, after a quarter of a century, the end of the beginning.
